# Specificity of Induction of Glycopeptide Antibiotic Resistance in the Producing Actinomycetes

**DOI:** 10.3390/antibiotics7020036

**Published:** 2018-04-25

**Authors:** Elisa Binda, Pamela Cappelletti, Flavia Marinelli, Giorgia Letizia Marcone

**Affiliations:** 1 Department of Biotechnology and Life Sciences, University of Insubria, via J.H. Dunant 3, 21100 Varese, Italy; elisa.binda@uninsubria.it (E.B.); pamela.cappelletti@uninsubria.it (P.C.); flavia.marinelli@uninsubria.it (F.M.); 2 The Protein Factory Research Center, Politecnico di Milano and University of Insubria, via J.H. Dunant 3, 21100 Varese, Italy

**Keywords:** actinomycetes, glycopeptide antibiotics, teicoplanin, A40926, *van* resistance genes

## Abstract

Glycopeptide antibiotics are drugs of last resort for treating severe infections caused by Gram-positive pathogens. It is widely believed that glycopeptide-resistance determinants (*van* genes) are ultimately derived from the producing actinomycetes. We hereby investigated the relationship between the antimicrobial activity of vancomycin and teicoplanins and their differential ability to induce *van* gene expression in *Actinoplanes teichomyceticus*—the producer of teicoplanin—and *Nonomuraea gerenzanensis*—the producer of the teicoplanin-like A40926. As a control, we used the well-characterized resistance model *Streptomyces coelicolor*. The enzyme activities of a cytoplasmic-soluble d,d-dipeptidase and of a membrane-associated d,d-carboxypeptidase (corresponding to VanX and VanY respectively) involved in resistant cell wall remodeling were measured in the actinomycetes grown in the presence or absence of subinhibitory concentrations of vancomycin, teicoplanin, and A40926. Results indicated that actinomycetes possess diverse self-resistance mechanisms, and that each of them responds differently to glycopeptide induction. Gene swapping among teicoplanins-producing actinomycetes indicated that cross-talking is possible and provides useful information for predicting the evolution of future resistance gene combinations emerging in pathogens.

## 1. Introduction

Glycopeptide antibiotics (GPAs) are drugs of last resort for treating severe infections caused by Gram-positive pathogens such as *Staphylococcus aureus* (SA), *Enterococcus* spp., and *Clostridioides difficile* [1]. Clinically important GPAs include first-generation vancomycin and teicoplanin—which, although discovered many decades ago, continue to be extensively used in clinical practice—and second-generation telavancin, dalbavancin, and oritavancin, which were recently approved for clinical use for their increased antimicrobial potency and superior pharmacokinetic properties [2,3,4]. Vancomycin and teicoplanin are natural product GPAs produced by soil-dwelling filamentous actinomycetes. Their common structural motif is a core heptapeptide scaffold containing aromatic amino acids that have undergone extensive oxidative cross-linking and decoration with different moieties, such as sugar residues, chlorine atoms and—in case of teicoplanin—a lipid chain. GPAs inhibit peptidoglycan (PG) synthesis by binding to the d-alanyl-d-alanine (d-Ala-d-Ala) terminus of the peptide stem of PG-precursor lipid II. The binding of GPAs to lipid II by forming five hydrogen bonds locks PG precursors, impeding subsequent cross-linking reactions [5,6]. Second-generation GPAs are semisynthetic derivatives of vancomycin- and teicoplanin-like molecules, where the chemical modifications were introduced outside the d-Ala-d-Ala binding pocket, mainly involving the appendage of hydrophobic aryl or acyl groups that mimic the natural lipid chain of teicoplanin. In fact, the superior antimicrobial potency of teicoplanin-like molecules is due to membrane anchoring of the hydrophobic tail, which strengthens the bond to membrane-localized lipid II [7,8]. Additionally, Dong et al. [9] demonstrated that lipidation is the key functional difference between vancomycin and teicoplanin related to their differing abilities of inducing a GPA resistance response in enterococci. More recently Kwun and Hong [10], using the harmless actinomycete *Streptomyces coelicolor* as a model resistance system, confirmed that teicoplanin-like derivatives are poor inducers of GPA resistance, and a lack of induction accounts for the susceptibility to these molecules.

Many different GPA-resistant phenotypes have been described in enterococci and staphylococci (for an extensive review, see Binda et al., 2014 [2]). In the two most prominent manifestations of resistance (VanA or VanB phenotypes), the GPA-induced expression of *van* genes remodels the bacterial cell wall. The replacement of the dipeptide (d-Ala-d-Ala) terminus of PG precursor with the depsipeptide d-alanyl-d-lactate (d-Ala-d-Lac) reduces by 1000-fold the GPA affinity to their molecular target [11]. Strains displaying this type of resistance are either resistant to both vancomycin and teicoplanin (VanA phenotype), or they are resistant only to vancomycin and susceptible to teicoplanin (VanB phenotype) [12]. Although the proteins directly involved in conferring VanA resistance—i.e., VanH, which converts pyruvate into d-lactate; VanA, a d-Ala-d-Lac ligase; and VanX, a d-Ala-d-Ala dipeptidase—are highly homologous to their counterparts in the VanB phenotype (VanHB, VanB, VanXB), the two-component regulatory systems controlling *van* gene transcription (VanS/VanR in VanA phenotype and VanSB/VanRB in VanB phenotype) are only distantly related [13]. In particular, the membrane-associated sensor domains of VanS and VanSB are unrelated in amino acid sequence, and they respond to GPAs by different mechanisms which account for the difference in induction specificity by vancomycin and teicoplanin and their semi-synthetic derivatives [13,14,15]. Many efforts [12,14,16,17] have been devoted to identifying the molecular species responsible for differently inducing VanS and VanSB, but their entity and mode of action—i.e., direct binding of the GPAs to the sensor domain or its activation by cell wall intermediates that accumulate as a result of antibiotic action—is still being questioned.

Since it is widely believed that GPA resistance mechanisms are ultimately derived from GPA-producing actinomycetes, which use them to avoid suicide during antibiotic production [2,14], in this paper we investigated the specificity of induction of GPA resistance in teicoplanin- and A40926-producing actinomycetes. Teicoplanin is produced by *Actinoplanes teichomyceticus*, and the teicoplanin-like molecule A40926 [18], which is the natural precursor of the second-generation dalbavancin, is produced by the recently classified *Nonomuraea gerenzanensis* [19,20]. As a control, we used the well-characterized resistance model *S. coelicolor*, which does not synthesize any GPA but does possess a *van* gene cluster conferring inducible resistance to vancomycin but not to teicoplanin, showing the features of the VanB phenotype [10,16,21]. The purpose of this study was to elucidate the relationship between GPA activity and the ability to induce *van* gene expression in the producing actinomycetes, which are considered the evolutionary source of resistance determinants emerging in pathogens, shedding light on the possible evolution of the *van* gene cluster.

## 2. Results and Discussion

Table 1 reports the minimal inhibitory concentrations (MICs) of vancomycin, teicoplanin, and A40926 against *S. coelicolor*, *A. teichomyceticus,* and *N. gerenzanensis* on solid media. MICs were measured on solid media, since the standard broth dilution method used in unicellular bacteria to determine MICs by turbidity is compromised in mycelial actinomycetes by the formation of multicellular aggregates and by the coexistence of cells in different physiological states (e.g., vegetative mycelium, aerial mycelium, and spores). This is particularly true for difficult-to-cultivate nonstreptomyces actinomycetes such as *Actinoplanes* and *Nonomuraea* strains, which form compact and different-sized clumps when growing in liquid cultures.

As expected, *S. coelicolor* was resistant to vancomycin and susceptible to teicoplanin and A40926. Its ∆*vanRS* mutant did not respond to vancomycin, and consequently, it was sensitive to it [10]. For these *Streptomyces* strains, MICs values were slightly higher than those previously reported in a liquid medium [10], which is likely due to the different cultivation method used. Interestingly, *S. coelicolor* and its ∆*vanRS* mutant were equally sensitive to bacitracin and ramoplanin, which are antibiotics structurally unrelated to GPAs that inhibit the late steps of PG synthesis by a diverse mode of action [22,23], evidently not mediated by VanS interaction.

*A. teichomyceticus* was resistant to all the GPAs tested (Table 1), although its chromosome harbours a canonical *vanHAX* gene cluster including the *vanRS* two component-regulatory system associated with the teicoplanin biosynthetic genes [24]. In this actinomycete, *van* genes are expressed constitutively, even in the absence of the antibiotic, making the cells intrinsically resistant to GPAs [24,25].

In contrast, the A40926-producing strain *N. gerenzanensis* was resistant to vancomycin (less than *A. teichomyceticus* and *S. coelicolor*) but sensitive to teicoplanin and to its own product, albeit at a lower extent. As previously described [26], *N. gerenzanensis* does not possess a *vanHAX* gene cluster, and the only known mechanism of resistance relies on the action of a VanY metallo-d,d-carboxypeptidase (named VanYn) that hydrolyses the C-terminal d-Ala residue of PG pentapeptide precursors. Interestingly, the well-characterized VanA and VanB type enterococci possess, in addition to *vanHAX* genes, an extra *vanY* gene that plays an ancillary unessential role in conferring glycopeptide resistance [27]. Upon vancomycin induction, enterococcal VanX cleaves any residual cytoplasmic d-Ala-d-Ala dipeptide, ensuring that the newly formed PG precursors terminate mostly in d-Ala-d-Lac, whereas VanY just acts on PG precursors that have escaped VanX hydrolysis and converts them into GPA-resistant tetrapeptides [27]. The integration of the pST30 plasmid containing the complete *vanRSHAX* gene cluster from *A. teichomyceticus* [25,28] into the *N. gerenzanensis* chromosome, consistently rendered the host strain more resistant to teicoplanin and A40926 in comparison to the parental strain harbouring only the *vanY* gene (Table 1), confirming the role of *vanHAX* genes in conferring high GPA resistance.

In contrast to *S. coelicolor*, both *A. teichomyceticus* and *N. gerenzanensis* were intrinsically resistant to both bacitracin and ramoplanin. This last result merits further investigation, although it is well-known that PG structure and density, and consequently antibiotic resistance profile, might dramatically vary among different genera of actinomycetes [19,29,30].

**Figure 1:**
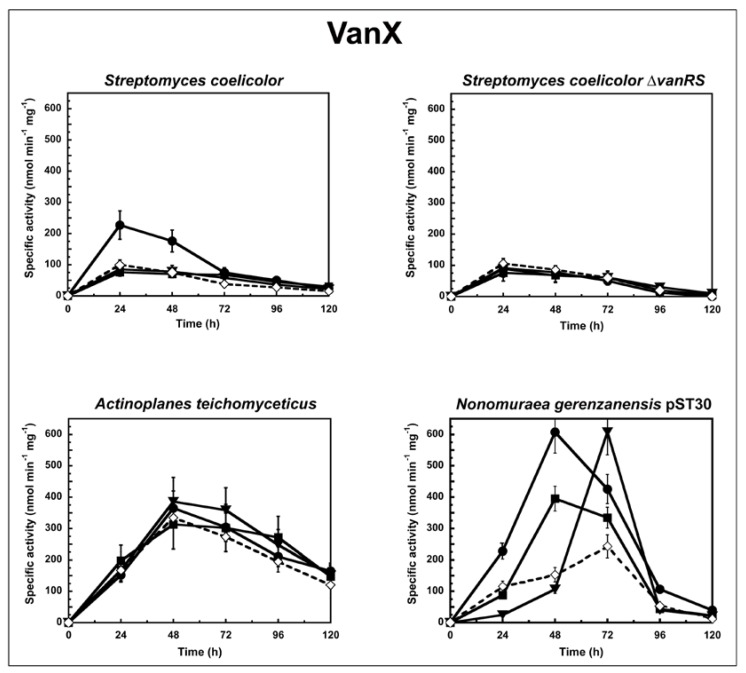
VanX activity in *S. coelicolor*, *S. coelicolor* Δ*vanRS*, *A. teichomyceticus*, and *N. gerenzanensis* pST30 grown in the absence (dotted line) or in the presence (continuous line) of subinhibitory concentrations of GPAs added at the moment of inoculum (see material and methods). VanX-specific activity is defined as the number of nanomoles of d-Ala released by the hydrolysis of d-Ala-d-Ala dipeptide at 37 °C per minute per milligram of protein contained in cytoplasmic fractions. Symbols represent non-induction (◇), induction with vancomycin (●), or teicoplanin (■), or A40926 (▼). The values represent the averages from three independent experiments, with a standard deviation of <5%.

**Figure 2:**
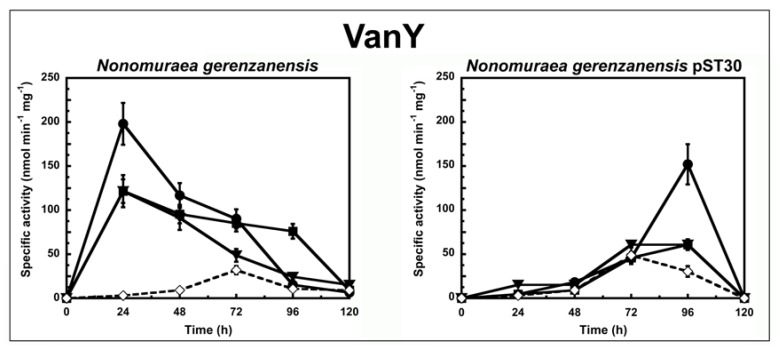
VanY activity in *N. gerenzanensis* and *N. gerenzanensis* pST30 grown in the absence (dotted line) or in the presence (continuous line) of subinhibitory concentrations of GPAs added at the moment of inoculum (see material and methods). VanY-specific activity is defined as the number of nanomoles of d-Ala released by the hydrolysis of N_ε_-acetyl-l-Lys-d-Ala-d-Ala tripeptide at 37 °C per minute per milligram of protein contained in membrane extracts. Symbols represent non-induction (◇), induction with vancomycin (●), or teicoplanin (■), or A40926 (▼). The values represent the averages from three independent experiments, with a standard deviation of <5%.

To determine the correlation between the antimicrobial activity of the GPAs and their ability to induce the *van* resistance system in actinomycetes, we followed the enzyme activities corresponding to either VanX or VanY peptidases in cells growing in liquid culture in the presence or absence of subinhibitory concentrations of GPAs (Figure 1 and Figure 2). The d,d-dipeptidase activity of VanX (Figure 1) and the d,d-carboxypeptidase activity of VanY (Figure 2) were measured by determining the amount of d-Ala released from the hydrolysis of the d-Ala-d-Ala dipeptide and of the N_ε_-acetyl-l-Lys-d-Ala-d-Ala tripeptide, respectively. d-Ala release was measured by using a d-amino acid oxidase coupled to a peroxidase [25,31]. As in the case of enterococci [27], VanX activity was detectable in the cytoplasmic fractions of *S. coelicolor*, *S. coelicolor* ∆*vanRS*, *A. teichomyceticus*, and *N. gerenzanensis* pST30 (Figure 1), and it was specific for the hydrolysis of d,d-dipeptides, being inactive on the ester d-Ala-d-Lac and on dipeptides substituted at the C or N terminus (data not shown). No VanX activity was detectable in *N. gerenzanensis* cytoplasmic fractions (see Table S1 Supplementary Material). Alternatively, VanY activity was detectable only in the membrane fractions of *N. gerenzanensis* and in its recombinant-derived *N. gerenzanensis* pST30 strain (Figure 2), consistent with the predicted VanY N-terminal structure, which contains a hydrophobic domain [31]. No VanY activity was detectable in *S. coelicolor*, *S. coelicolor* ∆*vanRS*, and *A. teichomyceticus* (see Table S1 Supplementary Material).

In *S. coelicolor* and *S. coelicolor* ∆*vanRS,* basal VanX activity (without any GPA addition) reached the maximum value within the 24 h of growth. When *S. coelicolor* parental strain was grown in the presence of subinhibitory concentrations of vancomycin (10 µg/mL), teicoplanin (0.75 µg/mL), and A40926 (0.75 µg/mL), only vancomycin induced an increase in VanX activity, and the level of enzyme activity doubled within the first 24 h from the induction (Figure 1). The addition of ramoplanin and bacitracin (both added at 0.45 µg/mL) did not show any effect on VanX activity (data not shown), thus indicating the specificity of vancomycin induction. Moreover, the addition of subinhibitory concentrations of vancomycin (in this case 0.6 µg /mL), teicoplanin (0.75 µg/mL), A40926 (0.75 µg/mL), and ramoplanin and bacitracin (both added at 0.45 µg/mL) to *S. coelicolor* ∆*vanRS* did not induce any increase in VanX activity, confirming the role of VanS in responding to vancomycin induction (Figure 1).

In *A. teichomyceticus*, basal VanX activity (without any GPA addition) reached the maximum value after 48 h of growth. No significant variation in VanX activity was detectable following the addition of 45 µg/mL of vancomycin, of 10 µg/mL of teicoplanin, or of 15 µg/mL of A40926 (Figure 1). As expected, the MICs of GPAs measured on *A. teichomyceticus* after 48 and 72 h of growth in the presence of teicoplanin were the same as reported in Table 1 (see Table S2 in Supplementary Material). These results are in agreement with the constitutive expression of *vanHAX* genes that, according to Beltrametti et al. [24], is due to an impaired phosphatase function of the mutated VanS, which is consequently locked in the “on” state in this organism [14]. The addition of ramoplanin and bacitracin did not exert any specific effect on the VanX activity in *A. teichomyceticus* (data not shown).

Interesting is what occurred in the recombinant strain *N. gerenzanensis* pST30 that, in addition to *vanYn*, also harbours the heterologous *vanX* from *A. teichomyceticus*. In this strain, the basal d,d-dipeptidase activity of VanX reached the maximum value after 72 h of growth. VanX activity was induced by 10 µg/mL of vancomycin, by 1.75 µg/mL of teicoplanin, and by 10 µg/mL of A40926, albeit with different kinetics and intensity (Figure 1). When the *A. teichomyceticus vanRSHAX* genes were integrated into the host genome, VanX activity was not longer under the control of the parental *vanRS* but became inducible by GPAs, undergoing regulation by a still-unknown circuit present in *N. gerenzanensis*, which responds to subinhibitory concentrations of vancomycin (within the 48 h of growth), A40926 (within the 72 h), and, albeit to less extent, teicoplanin (within the 48 h) (Figure 1).

To shed light on the specificity of induction of GPA resistance in *N. gerenzanensis*, we compared the VanY activity in *N. gerenzanensis* and in its recombinant strain *N. gerenzanensis* pST30 when grown in the absence or presence of GPAs (Figure 2). In both the strains, basal VanY activity reached its maximum activity after 72 h of growth. Upon induction with 10 µg/mL of vancomycin, 0.45 µg/mL of teicoplanin, or 2 µg/mL of A40926, VanY activity in the parental strain increased within the first 24 h (Figure 2), suggesting that VanY-based resistance was induced by the exposure to the three GPAs. As expected, MICs of GPAs measured on *N. gerenzanensis* after 48 and 72 h of growth in the presence of A40926 increased, as reported in Table S2 of Supplementary Material. Again, the addition of ramoplanin and bacitracin did not influence the activity profile (data not shown). Surprisingly, the membrane-associated VanY activity in *N. gerenzanensis* pST30 was significantly induced only by vancomycin, and its detection was delayed in comparison to the parental strain, reaching its maximum value after 96 h of growth (Figure 2). In the presence of A40926 and teicoplanin, the VanY activity was comparable to the basal level previously observed in the noninduced recombinant strain (Figure 2). Intriguingly, these data suggest that swapping heterologous *A. teichomyceticus* genes in *N. gerenzanensis* pST30 did alter the specificity of induction of the homologous VanY activity, although the heterologous VanX activity responded to induction by the three GPAs as the VanY activity did in the parental strain. It seems that in the presence of the heterologous VanX dipeptidase, homologous VanY carboxyesterase tends to play an auxiliary role as it occurs in VanA and VanB enterococci [21]. Irrespective of this finding, further investigations on the still-unknown regulatory genes controlling GPA resistance in *N. gerenzanensis* are needed to better explain these diverse responses to GPA induction. In a previous ‘*van* genes’ swapping experiment conducted by Hutchings et al., 2006, introducing the *Streptomyces toyocaensis* VanRS signal transduction system into *S. coelicolor* ∆*vanRS* switched inducer specificity to that of *S. toyocaensis*, whose resistant genes are induced by A47934 but not by vancomycin [21]. The authors concluded that the inducer specificity was determined by the origin of VanS/VanR [21]. More recently, Kilian et al. reported that the VanRS homologous two-component system VnlRS of *Amycolatopsis balhimycina* (which produces the vancomycin-type balhimycin) activated the transcription of the *vanHAX* genes in *S. coelicolor*, but not in *A. balhimycina* [32]. Surprisingly, the introduction of VnlRS from *A. balhimycina* into *S. coelicolor* induced teicoplanin resistance, most likely by activating further unknown genes required for teicoplanin resistance [32]. Although two members of a putative VanRS-like two-component signal transduction system were previously identified in the A40926 biosynthetic cluster in *N. gerenzanensis* [33], their role in controlling the VanY-based self-resistance mechanism and in responding to GPA induction is still unveiled and merit further investigations.

## 3. Materials and Methods

### 3.1. Strains and Media

*S. coelicolor* A3(2) was a gift from Mervyn Bibb, John Innes Institute, Norwich, UK [34]. The Δ*vanRS* mutant of *S. coelicolor* was generously donated by Hee-Jeon Hong, University of Cambridge, UK [21]. *Streptomyces* spp. strains were maintained as spores in 10% (*v/v*) glycerol and propagated in MS agar media [34]. Liquid media for streptomycetes were YEME [34] and BTSB (Difco). Colonies were picked up from agar plates and inoculated into 300 mL Erlenmeyer flasks containing 50 mL of YEME. Flask cultures were incubated on a rotary shaker at 200 rpm and 28 °C.

*N. gerenzanensis* ATCC 39727 [19], its recombinant strain *N. gerenzanensis* pST30 [25], and *A. teichomyceticus* ATCC 31121 [35] were maintained as lyophilized master cell banks (MCBs). The mycelium from the MCBs was streaked on slants of a salt medium (SM) [34] solidified with agar (15 g/liter). After its growth, the mycelium from a slant was homogenized in 10 mL of 0.9% (*w*/*v*) NaCl, inoculated into liquid SM, grown for 96 h at 28 °C with aeration, and stored as a working cell bank (WCB) in 1.5 mL cryovials at −80 °C. One vial was used to inoculate each 300 mL Erlenmeyer flask containing 50 mL VSP medium [26], and the flasks were incubated at 28 °C, with shaking at 200 rpm. Previous HPLC data showed that no A40926 or teicoplanin production occurred in this vegetative medium [26,35]. In the case of *N. gerenzanensis* pST30, the VSP medium was added with 50 µg/mL apramycin to maintain plasmid selection. Surface cultures were grown on V0.1 agar [26]. All medium components were from Sigma-Aldrich (St. Louis, MO, USA) unless otherwise stated.

### 3.2. Induction Experiments

*S. coelicolor* A3(2), its Δ*vanRS* mutant, *A. teichomyceticus* ATCC 31121, *N. gerenzanensis* ATCC 39727, and *N. gerenzanensis* pST30 were grown as described above. Vancomycin, teicoplanin, A40926, bacitracin, and ramoplanin (all from Sigma-Aldrich) were dissolved in MilliQ water and sterilized by filtration using 0.22 µm filters. Antibiotics were added to the cultures at the moment of inoculation using concentrations calculated as the half-point of MICs (see below and Table 1), except for vancomycin (10 µg/mL) in *S. coelicolor* A3(2), as reported by Kwun et al. [10]. At these concentrations, growth curves of noninduced and induced strains were overlapping.

### 3.3. MIC Determination

Cryovials of WCBs were thawed at room temperature and used to inoculate a VSP medium for *Nonomuraea* spp. and *Actinoplanes* spp. or YEME for *Streptomyces* spp. in the presence or the absence of GPAs. The strains were grown to exponential phase (approximately 72 h) at 28 °C with shaking. The mycelium was harvested by centrifugation, suspended in 0.9% (*w*/*v*) NaCl, and fragmented by sonication with a Vibracell Albra sonicator 400 W [26]. A suspension of sonicated hyphae (corresponding to 10^7^ CFU) was seeded onto V0.1 (*Nonomuraea* spp. and *Actinoplanes* spp.) or MS (*Streptomyces* spp.) agar plates supplemented with increasing concentrations of the following antibiotics: 0 to 100 µg/mL vancomycin in 10-µg/mL increments; 0 to 2 µg/mL teicoplanin in 0.1-µg/mL increments or 0 to 40 µg/mL teicoplanin in 10-µg/mL increments depending on the strain; and 0 to 5 µg/mL or 5 to 50 µg/mL A40926, depending on the strain, in 0.5-µg/mL or 2.5-µg/mL increments. The plates were dried and then incubated at 28 °C. MIC values were determined as the lowest antibiotic concentrations that inhibited visible growth after 10 days of incubation.

### 3.4. d,d-dipeptidase and d,d-carboxypeptidase Assays

Cells were harvested by centrifugation at 3600× *g* for 20 min at 4 °C, and then they were suspended in 2 mL of 0.9% (*w*/*v*) NaCl per gram of cells (wet weight). All of the following manipulations were conducted at 0 to 4 °C. The mycelium was fragmented by sonication with a Sonics Vibracell VCX 130. Sonication was carried out for 5 min on ice, with cycles of 30 s with an amplitude of 90% (90% of 60 Hz), with breaks of 10 s. The samples were then centrifuged at 39,000× *g* for 15 min, and the supernatants (cytoplasmic fractions) were collected. An alkaline extraction of the pellets (cell debris and membrane fractions) was carried out by adapting a protocol developed previously for extracting membrane-bound proteins [25,36]. The sedimented pellets were resuspended in ice-cold distilled water containing proteinase inhibitors (0.19 mg/mL phenylmethanesulfonyl fluoride and 0.7 µg/mL pepstatin, both purchased from Sigma-Aldrich), and then, immediately before centrifugation (28,000× *g* for 15 min at 4 °C), the pH was adjusted to 12 by adding an appropriate volume of 2.5 N NaOH. Immediately after centrifugation, the supernatants were neutralized to pH 7 by adding 0.5 M sodium acetate (pH 5.4). Enzymatic activities in the supernatant (cytoplasmic fractions) and in the resuspended insoluble fractions (membranes) were assayed as reported previously [36] by measuring the release of d-Ala from commercially available dipeptide (d-Ala-d-Ala, 10 mM; Sigma-Aldrich) and tripeptide (N_ε_-acetyl-l-Lys-d-Ala-d-Ala, 10 mM; Sigma-Aldrich). d,d-carboxypeptidase activity was confirmed using 10 mM UDP-MurNAc-l-Ala-d-Glu-*meso*-Dap-d-Ala-d-Ala (UK-BaCWAN, University of Warwick) as a substrate. The release of d-Ala was followed spectrophotometrically at 510 nm with a d-amino acid oxidase–peroxidase coupled reaction. Reaction mixtures contained 5 mM of the peroxidase colorimetric substrate 4-aminoantipyrine (Sigma-Aldrich), 3 U/mL d-amino acid oxidase (Sigma-Aldrich), 7.5 U/mL horseradish peroxidase (Sigma-Aldrich), and 6 mM phenol in 50 mM 1,3-bis[tris(hydroxymethyl)methylamino]propane (pH 7.4) in a final volume of 1 mL, as described in detail in [36]. To compare the d,d-dipeptidase and d,d-carboxypeptidase activities in the cytoplasmic and membrane extracts, the activity was expressed as the number of nanomoles of d-Ala released from the dipeptide or tripeptide per min per mg of protein in the extract.

## 4. Conclusions

By comparing the specificity of induction of VanX and VanY peptidases in three different soil-dwelling actinomycetes, we confirm that *S. coelicolor* has a VanB-phenotype responding to vancomycin but not to teicoplanin or to teicoplanin-like A40926. *S. coelicolor* does not produce any GPA but possessing resistance genes confers it a selective advantage since it shares the ecological niche (soil) with many other GPAs-producing actinomycetes. Interestingly, by a culture-independent approach using molecular probes, it has been recently estimated that the frequency of encountering a vancomycin-type producer in soil is from 2.5 to 5 times higher than for a teicoplanin-like producing actinomycete [37]. In contrast, the teicoplanin producer *A. teichomyceticus* is highly and constitutively resistant to all the GPAs, including its own product. On the contrary, lower resistance in *N. gerenzanensis* is induced either by vancomycin or teicoplanins, and is based on the action of a VanY carboxypeptidase, which in many enterococci and staphylococci plays an unessential and ancillary role in the presence of VanHAX system [27]. Evidently, although producing structurally similar GPAs, *A. teichomyceticus* and *N. gerenzanensis* do not share the same self-resistant mechanism. Previous comparative analyses of their biosynthetic GPA clusters indicated that many A40926 biosynthetic genes are more related to vancomycin-type genes than to their teicoplanin homologs [38]. The production of A40926 and teicoplanin are most likely the result of a convergent evolution rather than originating from the same common ancestor. Although the identity and the role of the putative VanRS-like two-component signal transduction system in *N. gerenzanensis* needs to be investigated, the results of gene swapping between *A. teichomyceticus* and *N. gerenzanensis* indicate that cross-talking of the two-component systems is possible, as previously demonstrated in other actinomycetes [21,32]. Determining the sequences and the protein structures of the putative VanRS two-component system in *N. gerenzanensis* may help to confirm that the inducer specificity is determined by the origin of VanRS proteins and may provide additional evidence on the role of GPAs as VanS effector ligands. Additionally, since the genes involved in GPAs resistance in pathogens have been recruited from the different antibiotic-producing actinomycetes, gene swapping among different GPA-producing actinomycetes is proving to be useful for unveiling the specificity of regulation and predicting the evolution of future resistance gene combinations emerging in pathogens.

## Figures and Tables

**Table 1:** Minimal inhibitory concentrations (MICs) of glycopeptide antibiotics (GPAs), ramoplanin and bacitracin. The values represent the average of the data from three independent experiments.

Actinomycetes	Vancomycin (µg/mL)	Teicoplanin (µg/mL)	A40926 (µg/mL)	Ramoplanin (µg/mL)	Bacitracin (µg/mL)
*S. coelicolor*	>100	1.5 ± 0.025	1.5 ± 0.075	0.9 ± 0.045	0.9 ± 0.05
*S. coelicolor* ∆*vanRS*	1.25 ± 0.03	1.5 ± 0.02	1.5 ± 0.02	0.9 ± 0.025	0.9 ± 0.035
*A. teichomyceticus*	90 ± 4.2	20 ± 1.5	32.5 ± 1.5	20 ± 1	20 ± 1.15
*N. gerenzanensis*	20 ± 1.6	0.9 ± 0.01	4 ± 0.2	20 ± 1.2	20 ± 1.3
*N. gerenzanensis* pST30	20 ± 1.05	2.75 ± 0.15	17.5 ± 0.875	20 ± 1.1	20 ± 1.2

## Supplementary Materials

The following are available online at http://www.mdpi.com/2079-6382/7/2/36/s1, Table S1: Maximum VanX and VanY activities during the growth (in the absence of GPAs) of *S*. *coelicolor*, *S*. *coelicolor* Δ*vanRS*, *A*. *teichomyceticus*, *N*. *gerenzanensis* and *N*. *gerenzanensis* pST30. The values represent the average of the data from three independent experiments, Table S2: MICs of GPAs in noninduced and teicoplanins-induced actinomycetes. The values represent the average of the data from three independent experiments.
