# Supplementary material for: Specificity of Induction of Glycopeptide Antibiotic Resistance in the Producing Actinomycetes
Source: Antibiotics (Basel). 2018 Apr 25;7(2):36. doi: 10.3390/antibiotics7020036 (PMC6022977; doi:10.3390/antibiotics7020036)
Supplement: Supplementary file 1 [file antibiotics-07-00036-s001.pdf]

Table 1 Supplementary Material. Maximum VanX and VanY activities during the growth (in the absence of GPAs) of *S. coelicolor*, *S. coelicolor ΔvanRS*, *A. teichomyceticus*, *N. gerenzanensis* and *N. gerenzanensis* pST30. The values represent the average of the data from three independent experiments.

	**Specific VanX activity (nmol min^{-1} mg^{-1})**	**Specific VanY activity (nmol min^{-1} mg^{-1})**
S. coelicolor	100±4.3	0
S. coelicolor ΔvanRS	106±5.1	0
A. teichomyceticus	334.35±12.91	0
N. gerenzanensis	0	31.91±0.85
N. gerenzanensis pST30	243.16±10.51	48.68±1.43

Table 2 Supplementary Material. MICs of GPAs in non-induced and teicoplanins-induced actinomycetes. The values represent the average of the data from three independent experiments.

	**Vancomycin (µg/ml)**		**Teicoplanin (µg/ml)**		**A40926 (µg/ml)**	
	**48h**	**72h**	**48h**	**72h**	**48h**	**72h**
Nonomuraea gerenzanensis	20±0.8	20±0.5	0.9±0.01	0.9±0.015	4±0.15	4±0.1
Nonomuraea gerenzanensis + 2 µg/ml A40926	32±0.9	32±0.8	1.2±0.03	1.2±0.02	5±0.2	5±0.25
Actinoplanes teichomyceticus	90±2.5	90±1.6	20±1	20±0.9	32.5±1.2	32.5±1.3
Actinoplanes teichomyceticus + 10 µg/ml teicoplanin	90±1.8	90±2.1	20±0.75	20±0.85	32.5±1.1	32.5±0.9
